# Establishment and Development of a National Newborn Screening Programme for Congenital Hypothyroidism in Turkey

**DOI:** 10.4274/Jcrpe.929

**Published:** 2013-05-30

**Authors:** Dilek Dilli, Sema Özbaş, Deniz Acıcan, Nergiz Yamak, Mustafa Ertek, Uğur Dilmen

**Affiliations:** 1 Dr. Sami Ulus Maternity and Children Research and Training Hospital, Department of Neonatology, Ankara, Turkey; 2 Turkish Directorate of Public Health, Ankara, Turkey; 3 Dr. Abdurrahman Yurtaslan Oncology Training and Research Hospital, Ankara, Turkey; 4 Yıldırım Beyazıt University Faculty of Medicine and Director General of Health Research, Department of Pediatrics and Neonatology Ministry of Health, Ankara, Turkey

**Keywords:** congenital hypothyroidism, newborn, screening programme, Turkey

## Abstract

**Objective:** To assess the Turkish National Newborn Screening Programme (NNSP) for congenital hypothyroidism (CH). Retrospective study based on the data from NNSP.

**Methods:** Since December 2006, a nationwide screening programme for CH has been conducted in Turkey by the Turkish Directorate of Public Health (TDPH) in cooperation with several institutions. We evaluated the database between January 2008 and July 2010 of this programme. According to the methodology of the NNSP, between three and five days of age (or at discharge from the hospital, if this occurs earlier) blood specimens were routinely collected from neonates on filter paper, by puncturing the heel. The accepted thyroid-stimulating hormone cut-off level for recall was 20 mU/L initially and 15 mU/L subsequently. The incidence of possible CH by years was reported.

**Results:** During the evaluation period, 3223765 newborns were tested. The mean annual incidence of possible CH showed a gradual increase over the years (1:888 in 2008, 1:592 in 2009, and 1:469 in 2010). Regional differences were noted. Although the mean age of blood sampling did not change by years, the mean age at notification for suspected CH decreased from 19.2 to 15.7 days from 2008 to 2010.

**Conclusions:** We reported the first assessment of NNSP in Turkey. An improvement in performance measures for the CH screening programme has been noted. Knowledge on incidence of confirmed CH is not yet available in the database.

**Conflict of interest:**None declared.

## INTRODUCTION

Detection and treatment of congenital hypothyroidism (CH) within the first few weeks of life will prevent the complications of this disorder. It is known that the clinical features of CH are often subtle and many newborns remain undiagnosed at birth (1). The slow development of obvious clinical symptoms, coupled with the importance of early treatment led to the implementation of newborn screening for CH (2).

Prior to the onset of newborn screening programmes, the incidence of CH, as diagnosed by its clinical manifestations, was in the range of 1:7000 to 1:10000 (3,4). The initiation of measurement of thyroid hormones and thyroid-stimulating hormone (TSH) in the cord blood paved the way for newborn screening, which was first introduced in 1974 using neonatal heel prick blood samples, a technique pioneered by Guthrie and Susi for phenylketonuria screening (4,5,6). With the advent of screening of newborn populations, the incidence of CH was initially reported to be in the range of 1:1000 to 1:4000 (2). With more experience gained from regional and national screening programmes, it has become apparent that the incidence varies by geographic location. According to the findings of the French newborn screening programme covering a 20-year period, the incidence of permanent hypothyroidism was 1:10000, whereas a report from the Greek Cypriot population over an 11-year period found an incidence of 1:1800 (7,8). In a study from the United States, it was reported that the incidence had increased from 1:4 094 in 1987 to 1:2372 in 2002 (9). The reason for the increased incidence is not clear, but one possible explanation may be a change in testing strategy. To identify newborns with CH for the purpose of early treatment, newborn screening programmes measure TSH or thyroxine (T4) concentrations, or both, in dried blood spots obtained from newborns. With increased sensitivity and accuracy of TSH methods, many countries around the world have switched from a primary T4-follow-up TSH approach to a primary TSH test. It is also a fact that more infants with milder CH will be detected if the TSH cut-off is lowered.

Screening programmes for CH have been developed in Canada, the United States, parts of Mexico, Western Europe, Japan, Australia, New Zealand, and Israel, and they are under development in parts of many countries in Eastern Europe, Asia, South America and Africa. Of the worldwide birth population of 127 million, it is estimated that 25 percent undergo screening for CH (10). In Turkey, because the health policies typically targeted a reduction in mortality and in morbidity from infectious diseases, although there were a few local programmes, there was no nationwide screening programme for CH until recent years. One of the basic requisites for a screening programme is the availability of epidemiological data regarding disease burden. Some pilot studies aiming to assess the epidemiology of CH showed a high CH prevalence in the country and paved the way for the initiation of a nationwide CH screening programme in Turkey, which was started on December 25, 2006 (11,12,13,14).

The purpose of this retrospective study was to assess the Turkish National Newborn Screening Programme (NNSP) for CH covering the time period between 2008 and 2010. We also aimed to evaluate whether there is an improvement from the beginning of the programme and to determine the encountered problems.

## METHODS

The screening programme for CH was started on December 25, 2006 by the Turkish Directorate of Public Health (TDPH), in cooperation with several institutions in Turkey. Two screening laboratories (in Ankara and in Istanbul) of the TDPH were involved in the programme. According to the programme schedule, the blood specimens were routinely collected between the 3rd and 5th days of life (or at discharge from the hospital, if this occurs earlier) on filter paper, by puncturing the heel. The samples are obtained in collaboration with the ongoing nationwide phenylketonuria and biotinidase deficiency screening programmes. The NNSP has been carried out in consultation with a scientific committee consisting of academicians from several universities in Turkey.

It has not been possible to access reliable data for the first year of the programme due to some technical problems. Therefore, we evaluated the database between January 2008 and July 2010.

**Blood Spot TSH Assay**

The filter paper cards are sent to two centralized laboratories of TDPH for testing on two days of the week. The samples are tested within three working days of receipt.

An enzyme-linked immunosorbent assay (ELISA) method is used at a cost of around 0.5 US$ for each examination (Neo-TSH ELISA Kit, Pishtaz Teb Zaman Diagnostics, Tehran, Iran). Controls provided with kits are run with each assay of samples and the results are validated only when the control values are found to be within the range specified by the manufacturer. Results are reported per volume of serum. At the beginning of NNSP, the cut-off point of TSH was 20 mU/L. However, a TSH value of 15 mU/L has been used as cut-off point since January 2009.

**Protocol for CH Screening**

**Figure 1:**
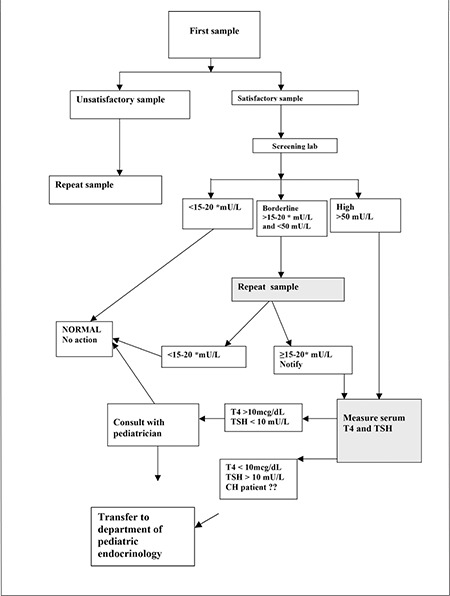
Algorithm for the notification of newborns with raised TSH on filter paper screening *TSH cut-off value was 20 mU/L in 2008, 15 mU/L between 2009 and 2010T4: thyroxine, TSH: thyroid-stimulating hormone, CH: congenital hypothyroidsm

Figure 1 shows the diagnostic algorithm used including the reporting and recall procedures advised by the Scientific Committee.

A filter paper sample which is not appropriately collected is defined as “unsatisfactory sample”. The sample taken in the first 48 hours of life is defined as “early sample”. A new blood specimen is requested from these newborns. For repeat samples, if the TSH is equal to or greater than the lower limit of the borderline range, notification automatically ensues. The first point of contact is the clinical coordinator (or designated deputy), requesting that the infant is recalled for confirmatory thyroid function tests. If the results are abnormal, the patient is transferred to the nearest department of pediatrics/pediatric endocrinology as suspected CH. The need for further diagnostic tests and the decision whether or not to treat the infant are at the clinician’s discretion. Information on the number of the patients in whom L-T4 therapy is started with a diagnosis of possible CH is obtained from the Province Health Directorates. These patients are followed up for three years to differentiate transient from permanent CH cases.

**Database and Statistics**

Data on NNSP for CH was obtained from TDPH. Between 2008 and 2010, a database was developed by Alpdata Software Company, who devised a program format comprising basic information (name, date of birth, date of sampling, address, change of address, gender, multiparity, prematurity, and neonatal TSH screening data).

All data were reviewed by the clinical team. Descriptive analyses were carried out.

**Ethics**

The parents were informed about NNSP. Heel-prick blood samples were collected from liveborn babies after prior written consent from the parents. The study was carried out with the written permission of the Scientific Committee of the TDPH.

## RESULTS

This retrospective study covered NNSP data for CH collected between January 2008 and July 2010. A total of 4848097 test results on 223765 newborns were evaluated. Male to female ratio was similar for all three years. Multiple birth rates were 1.3% in 2008, 1.6 % in 2009, and 1.7% in 2010.

Unsatisfactory sampling rates were 1.3%, 2.4%, 2.7%, respectively in the three study years. Early sampling rates were 24.1%, 27.4%, 28.4%. The results of early sampled specimens were not included in the study.

The numbers for unsatisfactory and early sampling, mean sampling date and mean age at notification are given in Table 1. Age at notification seems to gradually decrease over the years.

In the first test, a total of 83925 newborns were found to have borderline TSH elevations. During the study period, 6509 infants were found to have high TSH value. Table 2 gives information on the number of tested babies and first test screening results. The newborns with borderline TSH values in the first test (2.6% of the infants) were recalled to investigate the possibility of false negative screening results. In the repeat test, the TSH value was higher than the cut-off value in 10260 newborns (Table 3).

As previously stated, urgent notification was performed for newborns with high values at the first test. All infants with a TSH value higher that the cut-off point at the repeat test were referred to the clinician. Male to female rates among these cases were 1.04, 1.1 and 1.1, and multiple birth rates were 0.6%, 0.9% and 1.1% respectively by years.

During the study period, 16621 newborns were referred to the clinician. The number of infants in whom L-T4 therapy was started with a diagnosis of possible CH was significantly increased from 2008 to 2010 (Table 4).

The incidence rates of possible CH by 12 regions of Turkey between 2008 and 2010 were evaluated.

**Figure 2:**
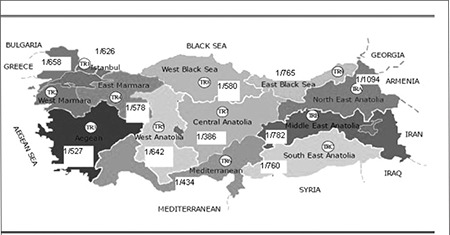
The incidence of possible CH by 12 regions of Turkey in 2009

It was noted that the highest incidence rates were in the Mediterranean and Central Anatolia regions throughout the study period. Figure 2 shows the incidence of possible CH by 12 regions of Turkey in 2009. 

## DISCUSSION

In this article, we described the early results of a NNSP for CH in Turkey, based on data recorded during a 2^1/2-y^ear period from 2008 to 2010. It seems that there is a high coverage, as the numbers screened are close to number of births in Turkey. The excess of tested babies over the number of liveborn babies reported by the Turkish Institute of Statistics may be explained by some infants being recorded and/or tested more than once, or these figures may be related to births taking place outside hospitals. Our evaluation has shown a favorable response to the neonatal screening programme and, in those infants with possible CH, reasonable mean ages (8.3 days) of repeat filter paper sample collection. It was noted that 2.08% of the samples were unsatisfactory. New blood specimens were requested from these newborns. We think that improvement in the training of the health personnel responsible for obtaining the samples may prevent unsatisfactory sampling.

The American Academy of Pediatrics (AAP) advocates the optimal testing time is between 48 hours and 4 days of age. However, screening before hospital discharge (for infants who are discharged early) or before transfusion is preferable to missing the diagnosis of CH. The goal of NNSP is to test babies between three and five days of life. However, the data indicate that 26.3% of the samples in this study were taken in the first 48 hours of life because of early discharge from the hospital. The number of tested babies was 3223765 during the 30-month study period. Turkey has 12 regions with different geographic and socio-cultural characteristics. Although the newborns tested within the first 48 hours of life were recalled for a second test, some may not have come back due to some traditional beliefs, socio-economical reasons, or difficulties in communication or transportation. Care providers therefore prefer to test all babies before discharge from the hospital for fear of missing CH cases. In the current study, we did not evaluate the results of early sampled specimens.

It is well known that unrecognized CH leads to mental retardation. Newborn screening and thyroid therapy started within two weeks of age can normalize cognitive development. In a research from Scotland, Ray et al (15) reported that the mean age of notification was 17.5-21 days before 1983, which was consistent with other reports from that time (16,17), and decreased to 10-11 days in 1993. In our screening programme, we noticed that age at notification gradually decreased from 2008 (19.2 days) to 2010 (15.7 days). We hope that it will continue to decrease by years.

According to recent data, with more experience from regional and national screening programmes, it has become apparent that the incidence varies by geographic location. In Turkey, the Western Black Sea, Eastern Anatolian, and the Middle Anatolian regions are reported to be iodine deficient areas with high goiter prevalence. There are a few pilot studies on prevalence of CH in Turkey performed before NNSP. Yordam et al (12) reported that the incidence of CH in the central part of Turkey (the Middle Anatolian region) was 1:2736 in 30097 newborns screened between 1991 and 1992. Similarly, Cinaz et al (14) reported the incidence of CH as 1:2525 in the same area between 2000 and 2007. The incidence figure reported for the Western Black Sea region of Turkey between 2000 and 2002 was 1:2326 (13). The NNSP data indicate that the incidence of possible CH cases significantly increased from 2008 to 2010 (1:888 in 2008, 1:592 in 2009, and 1:469 in 2010). This finding may be explained by the advent of a TSH-based screening programme with increased sensitivity and accuracy of method. Regional differences were noted for all years with the highest incidence of 1:250 in the Mediterranean region in 2010. The wide variability of CH incidence rates with the same methodology in different parts of the same country may reflect the degree of iodine deficiency in each region. However, there may be other factors than iodine deficiency affecting CH incidence in different regions. We believe that these factors should be identified. It can be easily seen that the given numbers will be lowered by the confirmation of the suspect cases after further evaluation. Nevertheless, the data presented in this paper may give some information about the incidence of CH in Turkey.

Nearly all screening programmes report a female preponderance, approaching a 2:1 female to male ratio (18). A report from Quebec showed this female preponderance occurs mostly with thyroid ectopy, and less so with agenesis (19). Recently, Zeinalzadeh and Talebi reported the results of screening for CH of 62459 infants in East Azerbaijan, Iran. Their recall rate was 2.5% with a CH prevalence of 1:666 (female:male ratio 1:1.4) (20). Evaluation of our screening programme did not show a female preponderance.

It was stated that the CH incidence nearly double in twin births (1:876) as compared to singletons (1:1765), and even higher with multiple births (1:575) (10). In the current study, multiple birth rate was also high among suspected CH cases.

Heel prick test for TSH or T4 or both may be used for CH screening. TSH screening has been shown to be more specific. Although T4 screening is more sensitive in detecting especially those newborns with rare hypothalamic-pituitary-hypothyroidism, it is less specific with a high frequency of false positives especially in premature infants (21). With increasing accuracy of TSH measurement, many screening programmes now carry out an initial TSH test to detect CH. In a mass programme, the recall rate with the TSH assay was approximately 0.05% (or 1:2000), or six times less than the 0.3% (or 3:1000) recall rate with the initial T4 approach (22). In Zeinalzadeh and Talebi’s study (20), the re-call rate was 2.5%. According to the data of NNSP, the recall rate was estimated as 1.9% in 2008 at a cut-off value of 20 mU/L. As expected, the recall rate increased to 2.8% and 3.8% in 2009 and 2010 when the cut-off value was lowered to 15 mU/L. 

Serum TSH and T4 levels need to be determined at recall in all infants with an abnormal or borderline TSH result on screening, before the start of any treatment. Most (60-70%) newborns suspected of CH because of elevated neonatal TSH levels have normal or nearly normal TSH and normal free T4 (fT4) after a few days, at the recall examination. Therefore, these newborns have very short-lasting hyperthyrotropinemia and are usually classified as “false positive” at CH screening, because the early neonatal abnormalities are attributed to a variety of transient causes that have no relevant adverse clinical consequences (23). In the study by Cinaz et al (14), of the 12626 newborns, 1136 were found to have high TSH levels and were recalled for T4, fT4 and TSH tests. According to their results, 45 newborns were found to require follow-up, and after a three-year follow-up, 27 had hyperthyrotropinemia and 5 had persistant CH (3 with compensated hypothyroidism) (14). In Zeinalzadeh and Talebi’s study, the rate of unconfirmed CH prevalence was 1:666. We also observed that the number of the cases with borderline TSH elevation in the first test had decreased from 838925 to 10260 in the repeat test. Although of a total of 16621 newborns referred to the clinician only 4966 were diagnosed to be possible CH cases, with an average incidence of 1/650, the three-year follow-up results of these cases are not available yet.

Screening is not a confirmatory diagnosis and requires further investigations. Identification of primary hypothyroidism, which is the focus of newborn screening for CH, is reflected by elevated TSH and low serum fT4 concentrations. Transient hypothyroidism also involves an initially elevated TSH concentration and low or normal T4 level, but subsequent measurement reveals normal T4 and TSH concentrations. Guidelines formulated by several professional organizations exist for the medical management of CH and begin with serum thyroid-function tests to confirm the diagnosis according to established algorithms (24). Recently, Donaldson et al (25) stated key recommendations in the management of CH; these include: 1) screening on day 3 so that severely affected infants can begin treatment within the first 10 days of life; 2) setting the TSH referral cut-off at 8-10 mU/L; 3) adopting a disciplined diagnostic algorithm to evaluate referred cases, with measurement of venous fT4; 4) to conduct TSH and thyroglobulin measurements in combination with dual ultrasound and radioisotope imaging; 5) initial treatment with a T4 dose of 50 µg daily in infants weighing ≥2.5 kg and 15 µg/kg/day in infants weighing < 2.5 kg, followed by weekly review until thyroid function is normalised; and 6) maintenance of fT4 levels between 15-26 pmol/L and TSH between 0.5-5 mU/L thereafter. 

In Turkey, according to the algorithm used by NNSP, infants detected with abnormal thyroid screening tests were recalled immediately for examination, and a venipuncture blood sample was obtained for confirmatory serum testing. These infants were referred to the nearest department of pediatric endocrinology, if needed. Rarely, the newborn screening test results can be in error, or human error can result in failure to notify the infant’s physician of abnormal test results. In this context, the AAP and the American Thyroid Association advise that physicians cannot and must not relinquish their clinical judgment and experience in the face of normal newborn thyroid test results. They state that when clinical symptoms and signs suggest hypothyroidism, regardless of newborn screening results, serum fT4 and TSH determinations should be performed (26).

Finally, it must be appreciated that NNSP for CH in Turkey has improved since the beginning of the programme. The incidence of CH is notably higher in Turkey than reported in most other countries. Iodine deficiency and/or dyshormonogenesis or other factors might contribute to this high incidence. Data on the incidence of ‘confirmed CH’ cases are not yet available in the database and this is a limitation of this screening programme. Currently, those responsible for the screening programme are in the process of recording the final results of these patients. Our future goal is to reach the final results of all infants screened since 2007 and also to contact the endocrinologists who are in collaboration with the screening center. To differentiate true cases of CH from cases of transient hypothyroidism, we should know the long-term follow-up results of suspected cases. We plan to publish these data in a future paper.

**Acknowledgement**

We would like to express our sincere thanks to all members of the Scientific Committee for their significant contribution in the establishment and development of NNSP in Turkey. The skillful technical assistance of all laboratory workers of Turkish Directorate of Public Health is greatly appreciated.

* The Members of the Scientific Committee of the Turkish National Newborn Screening Programme (in alphabetical order):

Abdullah Bereket; Marmara University Faculty of Medicine, Department of Pediatric Endocrinology, İstanbul, Turkey

Rüveyde Bundak; İstanbul University Faculty of Medicine, Department of Pediatric Endocrinology, İstanbul, Turkey

Peyami Cinaz; Gazi University Faculty of Medicine, Department of Pediatric Endocrinology, Ankara, Turkey

Yahya Laleli; Düzen Laboratory, Ankara, Turkey

Behzat Özkan; Association of State Hospitals, General Secretary of the South District of İzmir, İzmir, Turkey

Z. Alev Özön; Hacettepe University Faculty of Medicine, Department of Pediatric Endocrinology, Ankara, Turkey

Doğan Yücel; Ankara Education and Research Hospital, Department of Biochemistry, Ankara, Turkey

## Figures and Tables

**Table 1:**
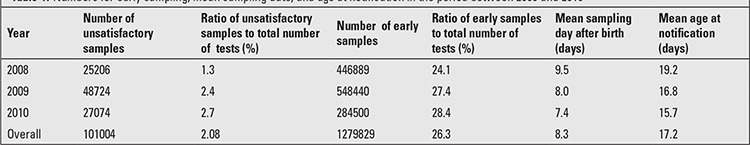
Numbers for early sampling, mean sampling date, and age at notification in the period between 2008 and 2010

**Table 2:**
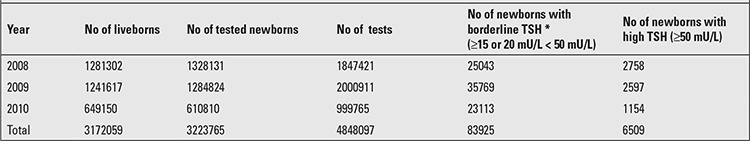
The number of liveborn babies, tested newborns, tests carried out, and thyroid-stimulating hormone (TSH) elevation at first screening are given between January 2008 and July 2010

**Table 3:**
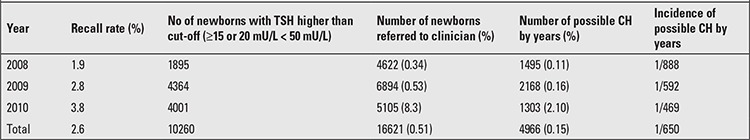
Recall rate according to borderline thyroid-stimulating hormone (TSH) elevation at first test, number of newborns with a TSH value of higher than cut-off point at repeat test, number of newborns referred to clinician, rate of newborns referred to clinician, and incidence of possible congenital hypothyroidism (CH) by years

**Table 4:**
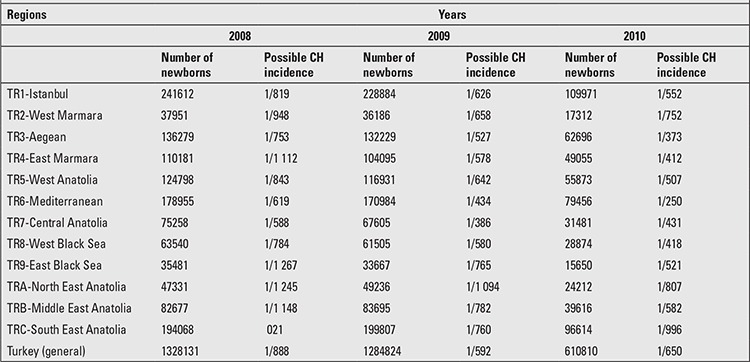
The incidence of possible congenital hypothyroidism (CH) by 12 regions of Turkey between 2008 and 2010
